# The Regulation of Lipokines by Environmental Factors

**DOI:** 10.3390/nu11102422

**Published:** 2019-10-11

**Authors:** Diego Hernández-Saavedra, Kristin I. Stanford

**Affiliations:** 1 Dorothy M. Davis Heart and Lung Research Institute, The Ohio State University Wexner Medical Center, Columbus, OH 43210, USA; diego.hernandez-saavedra@osumc.edu; 2 Department of Physiology and Cell Biology, The Ohio State University Wexner Medical Center, Columbus, OH 43210, USA

**Keywords:** type 2 diabetes, obesity inflammation, exercise, lipokines

## Abstract

Adipose tissue is a highly metabolically-active tissue that senses and secretes hormonal and lipid mediators that facilitate adaptations to metabolic tissues. In recent years, the role of lipokines, which are lipid species predominantly secreted from adipose tissue that act as hormonal regulators in many metabolic tissues, has been an important area of research for obesity and diabetes. Previous studies have identified that these secreted lipids, including palmitoleate, 12,13-diHOME, and fatty acid–hydroxy–fatty acids (FAHFA) species, are important regulators of metabolism. Moreover, environmental factors that directly affect the secretion of lipokines such as diet, exercise, and exposure to cold temperatures constitute attractive therapeutic strategies, but the mechanisms that regulate lipokine stimulation have not been thoroughly reviewed. In this study, we will discuss the chemical characteristics of lipokines that position them as attractive targets for chronic disease treatment and prevention and the emerging roles of lipokines as regulators of inter-tissue communication. We will define the target tissues of lipokines, and explore the ability of lipokines to prevent or delay the onset and development of chronic diseases. Comprehensive understanding of the lipokine synthesis and lipokine-driven regulation of metabolic outcomes is instrumental for developing novel preventative and therapeutic strategies that harness adipose tissue-derived lipokines.

## 1. Introduction

Diabetes and obesity share several etiological features and continue to rise at epidemic rates despite the continued healthcare efforts toward prevention and treatment. There are multiple factors that contribute to the development of type 2 diabetes (T2DM) including genetic factors and environmental stimuli, such as diet, lifestyle, and even atmospheric conditions (temperature). These various environmental factors elicit important metabolic adaptations in several organisms through a variety of nutrient, energy, and thermoregulatory mechanisms [1,2,3,4,5], which can affect the physiological response. The coordination of multiple cellular systems that allows them to respond to environmental stimuli is vital to a successful adaptive response.

Whole-body metabolic homeostasis is achieved through the concerted effort of several tissues including liver, skeletal muscle, and adipose tissue, which account for the uptake and metabolism of glucose and other metabolites. Adipose tissue is a significant contributor to metabolic adaptations in disease development as well as disease prevention. Cellular dysregulation of adaptive nutrient-sensing mechanisms in adipose tissue can lead to the onset of several chronic diseases. The enhancement of adaptive processes is vital for improved metabolic adaptations and overall health [6]. Additionally, environmental factors including exercise and cold exposure elicit a robust and differential metabolic adaptation in white (WAT) and brown adipose tissue (BAT) in mice and humans [6,7,8,9,10,11,12,13]. Thus, the metabolic flexibility of adipose tissue is essential in contributing to the tissue’s protective response.

## 2. Adipose Tissue

Adipose tissue can be broadly divided into two different types including white adipose tissue (WAT) and brown adipose tissue (BAT). Each of these depots have distinct morphological, physiological, and metabolic features. White adipocytes have unilocular fat droplets and store large amounts of triglycerides. The presence of WAT in the visceral compartment has been associated with T2DM and insulin resistance in both rodents and humans [14,15,16]. Conversely, brown adipocytes have multilocular lipid droplets [17,18], and increased mitochondrial density and energy metabolism [19]. Another important difference between adipose tissue depots resides in the size disparities that exist between brown and white adipose tissue depots, where BAT accounts for around 1.5% of total body mass and only 4.3% of total fat mass [20,21]. Despite the smaller proportion of BAT-to-WAT, BAT displays greater metabolic activity compared to WAT and an increase in BAT activation or mass are both associated with improvements in glucose tolerance and insulin sensitivity [6,21,22,23].

Adipose tissue also functions as a complex and highly dynamic endocrine organ. Adipose tissue actively secretes bioactive signals that modulate metabolic homeostasis, neuroendocrine function, and immune function [24,25,26,27]. One class of these bioactive signals are termed lipokines, and are defined as lipid molecules derived from adipose tissue that can coordinate a wide array of cellular processes.

## 3. Lipokines

In addition to the physiological and morphological differences in adipose tissue depots, cellular lipids comprise a wide variety of families and species that carry out diverse functions within the cell. This includes functions such as cell signaling (eicosanoids, phosphoinositides, endocannabinoids, sphingosine, lipokines, etc.), which serve as structural components of membranes (sphingolipids, cholesterol, cardiolipins, etc.), and bioenergetic substrates and mediators (fatty acids, cardiolipins, acylcarnitines, etc.) [28]. Among the signals that can originate from adipose tissue, lipokines have demonstrated an abundant therapeutic potential by mediating the communication between adipose tissue and other metabolic tissues [29,30,31,32,33]. Lipokines including insulin-sensitizing Palmitoleate (C16:1n7) [32,34] and the exercise-induced and cold-induced 12,13-diHOME [30,31], are important for understanding the inter-tissue communication that either prevents or leads to disease. Certain species of the newly described fatty acid–hydroxy–fatty acids (FAHFAs) [33,35] have great therapeutic potential in restoring glucose homeostasis. Adipose-derived lipokines are important mediators of whole-body cellular homeostasis, metabolism, stress, and inflammation.

In this paper, we will explore the genetic and environmental regulation of lipokines and their therapeutic properties in multiple targets. We will provide a comprehensive view of the literature regarding these novel lipokines and untangle the controversies surrounding these molecules. Lastly, we will explore their clinical translatability on the basis of disease susceptibility, which may lead to the development of clinical interventions.

### 3.1. Origin and Function of Lipokines

The ability of lipid species to modulate metabolic processes has been known for many decades, but the term “lipokine” has a relatively recent origin. First defined by the Hotamisligil lab outlining lipid species that act as endocrine factors in 2008 [32,34], the term lipokine distinguishes them from other lipids that have only structural or energetic functions. Since then, studies have identified several lipokines that are all structurally different but are able to elicit robust effects on metabolic homeostasis. Lipokines were initially described as important lipid products that emanate from adipose tissue (AT), but later studies indicated that these lipid products can also be produced from other metabolic tissues [36,37,38]. For the purpose of this review, we will focus on the main site of production, WAT and BAT, and refer to lipokines as AT-derived lipid compounds with endocrine activity.

### 3.2. Palmitoleate (C16:1n7)

#### 3.2.1. Discovery, Structure, and Synthesis of Palmitoleate

Fatty acids have distinct metabolic effects. Saturated fatty acids (SFAs) can lead to insulin resistance, while mono-unsaturated and polyunsaturated fatty acids (MUFAs and PUFAs) have either no effect on insulin resistance [39] or can improve insulin sensitivity [39,40]. Studies have identified an MUFA and palmitoleate (PAO), as a lipokine, which expands the role of fatty acids from signaling lipids into long-distance hormonal regulators [32,34].

The lipokine PAO (C16:1n7) was first discovered in fatty-acid binding protein 4/5 (FABP4 and FABP5) deficient mice, which display increased insulin sensitivity compared to wild type mice [41]. PAO or (9Z)-hexadec-9-enoic acid, possesses an unusual unsaturation on carbon 7 and can be largely found in AT triglycerides in a cis-configuration or trans-configuration. PAO is a product of de novo lipogenesis in adipose tissue, and deletion of FABP4/5 in adipose tissue increases de novo synthesis of PAO by upregulating the lipogenic genes *Fas* (FAS, Fatty acid synthase) and *Scd1* (SCD1, Steroyl-CoA desaturase 1) [32]. FABP4/5 deficiency promotes PAO (C16:1n7) accumulation in different lipid portions such as free fatty acids (FFA), diacylglycerol (DAG), and triglycerides (TAG) in adipose tissue [32,34]. Supplementation with plasma lipids from adipose-FABP4/5 deficient mice, which are rich in PAO, into isolated myotubes in vitro enhanced insulin signaling, and intralipid infusions enriched with PAO resulted in increased expression of the insulin receptor (IR), and the phosphorylation of insulin receptor substrates (IRS1, 2) and Protein kinase B (AKT) in liver and muscle. The addition of PAO alone reduced SCD-1 activity in rat hepatoma FAO cells [32], which contributes to the decreased hepatic steatosis observed in liver of FABP-deficient mice [42]. Overall, enhanced PAO synthesis and secretion by adipose tissue robustly decrease hepatic lipogenesis and increase skeletal muscle insulin action in vitro. These actions identify a role of PAO as a mediator of inter-tissue metabolism.

**Figure 1:**
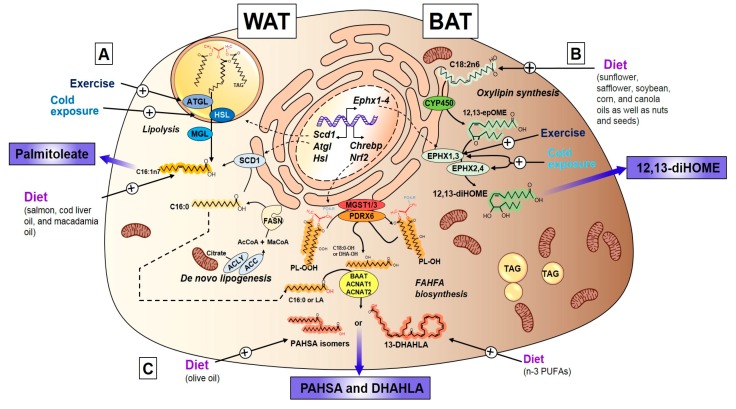
Lipokine synthesis in WAT and BAT. Bioactive lipids termed lipokines are synthesized primarily in adipose tissue from white (WAT) and brown (BAT) adipose depots. (**A**) Palmitoleate can be synthesized in WAT from de novo lipogenesis, starting with the cleavage of mitochondrial citrate by ACLY, followed by the rate limiting enzymes ACC and FASN, generating palmitate or palmitic acid, and further desaturated by Stearoyl-CoA Desaturase (SCD1) into palmitoleate. Palmitoleate can also be obtained from sympathetic-induced, exercise-induced, and cold-induced lipolysis (HSL, ATGL, and MGL) of triglycerides (TAG), which generates the diacylglycerol (DAG) or free fatty acid (FFA) form of palmitoleate. Lastly, palmitoleate can be obtained directly from the diet and provide a bioactive form of cis-palmitoleate or trans-palmitoleate. (**B**) 12, 13-diHOME (12,13-dihydroxy-9Z-octadecenoate) lipokine is primarily synthesized from BAT from essential fatty acid linoleic acid provided in the diet. The first synthetic step involves CYP 450 group of enzymes that give rise to the epoxide 12, 13-epOME, and is hydrolyzed by ER-residing or cytosolic EPHX1/3 or EPHX2/4, respectively. The final hydrolysis step generates 12, 13-diHOME. Both exercise and cold exposure are strong activators of the last step (*Ephx1-4*) of 12, 13-diHOME synthesis. (**C**) Fatty acid hydroxy fatty acid (FAHFAs) constitute a novel lipid class that act as lipokines including 5-PAHSA, 9-PAHSA, and 13-DHAHLA. FAHFA synthesis is thought to start with the modulation of oxidative stress by Nrf2 and de novo lipogenic factor Carbohydrate Response Element Binding Protein (ChREBP), which regulate the peroxide formation and synthesis of fatty acids. The first step involves the generation of a phospholipid peroxide substrate obtained from membrane residing phospholipids spontaneously oxidized or generated by flavin-containing monooxygenases. The first committed step involves the conversion of the peroxide into a phospholipid with the hydroxy fatty acid by GSH-requiring peroxidases MGST1/3 and PDRX6. This hydroxy-fatty acyl is released from phospholipid and then coupled to an Acyl-CoA generated in de novo lipogenesis, (i.e., palmityl-CoA). This is completed by the action of acyl transferases ACNAT1, ACNAT2, and BAAT. This last step creates FAHFAs with either isomers of PAHSA or DHAHLA. ACLY: ATP-citrate lyase. FASN: Fatty Acid Synthase. ACC: Acetyl-CoA Carboxylase. SCD1: Stearoyl-CoA Desaturase. HSL: Hormone-sensitive lipase. ATGL: Adipose tissue triglyceride lipase. MGL: Monoglyceride lipase. CYP 450: Cytochrome P450. EPHX1-4: Epoxide Hydrolase 1-4. GSH: Glutathione. MGST1/3: Microsomal Glutathione S-Transferase 1/3. PDRX6: Peroxiredoxin 6. ACNAT1/2: Acyl-coenzyme A amino acid N-acyltransferase 1. BAAT: Bile acid-CoA: amino acid N-acyltransferase.

PAO is one of the most abundant fatty acids in serum and adipose tissue [43]. PAO can be endogenously synthesized in adipose tissue and contribute to its accumulation in specific lipid moieties. De novo generation of PAO begins with mitochondrial citrate accumulation and export into the cytosol, which leads to the polymerization and activation of rate-limiting enzyme Acetyl-CoA Carboxylase (ACC). This increases Malonyl-CoA. Next, FAS adds Acetyl-groups to Malonyl-CoA, which generates Palmitate (C16:0) in the cytosol. This is followed by the desaturation by SCD1 within the ER into the monounsaturated fatty acid (MUFA) and palmitoleic acid (Figure 1). SCD1 can also desaturate stearate into oleate, which also contributes to metabolic homeostasis [37,38]. Moreover, de novo lipogenesis in adipose tissue is regulated by the rate limiting steps ACC and FAS, which reside in the endoplasmic reticulum (ER) and cytosol, respectively (Figure 1). The rate of PAO synthesis is regulated by both the synthesis and desaturation of palmitate. Importantly, trans-palmitoleic acid, which was previously thought to be obtained only from the diet, can be endogenously generated in the liver by the cleavage of trans-vaccenic acid (trans-C18:1 n-7) by hepatic peroxisomal β-oxidation [44], which produces palmitoleic acid. This novel hepatic retro-conversion mechanism of an abundant dietary fatty acid (trans-vaccenic acid) leads to the accumulation of PAO in multiple tissues [44]. Mice deficient in adipose tissue Transcriptional repressor B cell lymphoma 6 (*Bcl6*) have increased de novo lipogenesis and an accumulation of PAO in the TAG of serum and adipose tissue. These mice are also more sensitive to insulin and have enhanced glucose uptake in liver and other tissues [45], which further defines a role for PAO to affect metabolism in the liver, muscle, and adipose tissue.

PAO can be obtained from different dietary sources contributing to the accumulation of PAO in the body. Dietary sources of PAO include salmon (6% of total lipids), cod liver oil (7% of total lipids), olive oil, chocolate, eggs, macadamia nut oil (*Macadamia integrifolia*) (∼17% of total lipids), and sea buckthorn with the highest reported concentration in its pulp (32%–42%) [46]. It can also be synthesized by African oil palm protoplasts (*Elaeis guineensis*) with ~30% of total lipids [47]. These data indicate that an increase in PAO resulting from either dietary intake or de novo synthesis may contribute to improvements in metabolic homeostasis.

Structurally, PAO can be found in a cis-configuration (C16:1 c9) or trans-configuration (C16:1 t9), and both forms are associated with improvements in insulin sensitivity and glucose homeostasis [32,48,49]. Trans-PAO can be found in many dairy products and ruminant trans fats [43,50]. Despite the high content of C16:1n7 in certain oils, bioavailability, absorption, and conversion of PAO remains poorly studied. It is now widely accepted that dietary PAO significantly contributes to PAO levels in the body and that dietary enrichment could contribute to its bioaccumulation (Figure 1). This may be necessary to improve metabolic homeostasis. Thus, with the discovery and identification of the first adipose-specific lipokine PAO, a link was established for long-range inter-tissue communication in which products of de novo lipogenesis can act as endocrine factors that modulate metabolic adaptation.

#### 3.2.2. Endocrine Action of Palmitoleate

**Figure 2:**
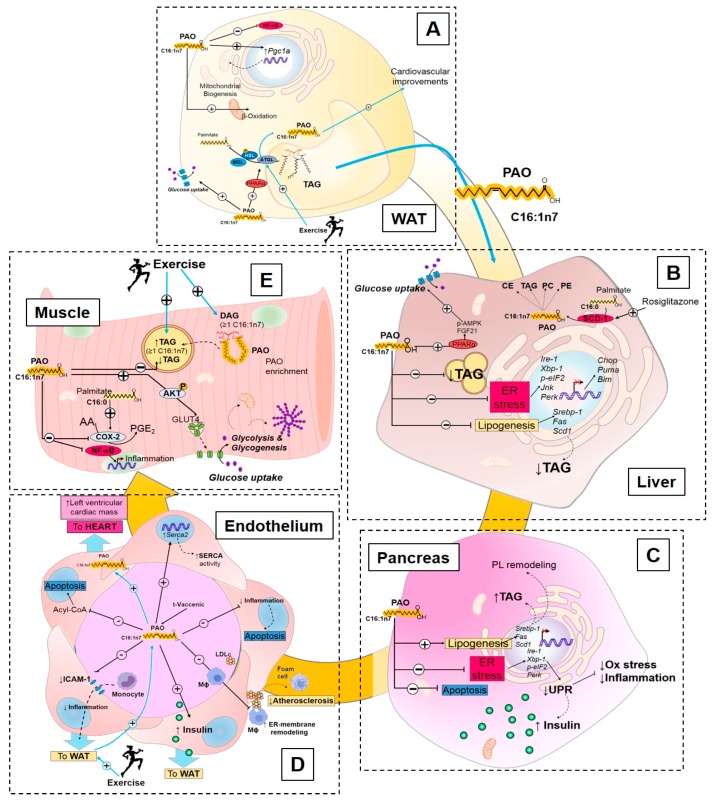
Endocrine effects of Palmitoleate. (**A**) Palmitoleate (PAO) is synthesized and stored in adipose tissue in TAG. Upon lipolytic stimulation (exercise, in blue), PAO is released and secreted to act in an autocrine or endocrine way. PAO can act in adipose to decrease inflammation (NF-kB), increase mitochondrial biogenesis (Pgc-1a), and β-oxidation. PAO can increase lipolysis and improve glucose uptake. (**B**) In the liver, PAO can directly stimulate glucose uptake through an AMPK/FGF21 mechanism. It can reduce steatohepatitis by reducing lipogenic gene expression and decrease endoplasmic reticulum (ER)-stress. In liver, insulin sensitizer rosiglitazone enhances desaturation and PAO enrichment in cholesteryl-ester (CE), TAG, and phospholipid portions (PC and PE). (**C**) In pancreatic β-cells, PAO leads to lipogenesis activation, decreased ER-stress, and apoptosis, which contribute to the improvement of β-cell endocrine function. Increase lipogenic genes in β-cells leads to phospholipid remodeling that results in pancreatic protection and insulin release. (**D**) In endothelial cells, PAO improves ER pump Sarcoplasmic/Endoplasmic Reticulum Calcium ATPase 2 (*Serca2*) expression and SERCA activity, which decreases Acyl-CoA accumulation and inflammation that leads to reduced apoptosis. PAO improves macrophage (Mϕ) membrane-lipid remodeling and reduced atherosclerosis. PAO can also enhance insulin delivery to WAT and decrease monocyte adhesion and recruitment to AT. Exercise (in blue) leads to AT lipolysis and PAO secretion, which, in turn, contributes to PAO delivery to the heart and contributes to cardiometabolic improvements. (**E**) PAO reduces inflammation and oxidative stress in skeletal muscle, and, through AKT phosphorylation, it improves glucose uptake. PAO also leads to reduced intramyofibrillar TAG accumulation, and exercise (in blue) contributes to PAO enrichment in TAG and DAG in muscle.

Enrichment of PAO has been associated with improvements in insulin resistance and glucose tolerance [32,34,49,51]. Circulating PAO can act in different peripheral tissues or in an autocrine manner in adipose tissue to contribute to the overall beneficial effects on metabolic health, including fatty acid synthesis and accumulation, glucose homeostasis, and stress responses (Figure 2).

##### Palmitoleate Modulates De Novo Lipogenesis

Adipose tissue is a dynamic endocrine tissue that can contribute to PAO secretion and, in turn, feedback to WAT, and can modulate metabolic homeostasis. PAO is one of the most abundant FA in WAT and, under fasting conditions, serum and WAT adipose PAO concentrations are highly correlated [43,52,53,54]. Secreted PAO acts in an autocrine manner to stimulate WAT and increase lipolysis via PPARα to activate adipose tissue triglyceride lipase (ATGL) and hormone-sensitive lipase (HSL) phosphorylation (pSer660-HSL) [55]. Loss of the lipolytic enzyme HSL decreases FA synthetic enzymes SCD1 and 2. Elongation of very long-chain fatty acids protein 3/5/6 (ELOVL3, 5, and 6), which lowers PAO incorporation into TAG from WAT, serum NEFA, and serum TAG [56]. This underscores the importance of the specific effects of PAO on FA synthesis, uptake, and enrichment in adipose tissue leading to autocrine and paracrine effects that can contribute to improved glucose homeostasis (Figure 2A).

In the absence of chaperones FABP4/5, PAO secreted from WAT acts in an endocrine manner signaling to the liver to decrease lipogenesis and reduce hepatosteatosis [32]. In the mouse model of spontaneous obesity and T2DM KK-Ay, administration of PAO (300 mg/kg of palmitoleic acid) protected the mice against hyperglycemia and hypertriglyceridemia, decreased the expression of hepatic lipogenic genes (Sterol regulatory element-binding protein 1 [*Srebp-1*], *Fas*, and *Scd1*), and lowered TAG accumulation in the liver [57] (Figure 2B). PAO also acts in an endocrine manner by stimulating pancreatic lipogenesis and enhancing its endocrine function. PAO increases pancreatic β-cell phospholipid remodeling and TAG synthesis, and promotes cell viability and mitogenesis [58]. Thus, PAO displays tissue-specific lipogenic effects that positively impact the tissue function.

##### Palmitoleate Improves Glucose Homeostasis and Insulin Sensitivity

PAO promotes insulin sensitivity in adipose tissue. Exogenous PAO directly stimulates lipolysis, fatty acid esterification, and mitochondrial β-oxidation, and increases oxygen consumption, and Glucose transporter 4 (GLUT-4) translocation, which contribute to WAT glucose homeostasis [55,59,60] (Figure 2). In healthy humans, subcutaneous adipose tissue is correlated to increased rates of insulin sensitivity [61,62,63,64]. Subcutaneous adipose tissue in the gluteofemoral region significantly contributes to the circulating PAO pool [65]. Gluteofemoral subcutaneous adipose has increased SCD1 activity, which may be partly responsible for the protective metabolic effects [65]. The greater FA desaturation by SCD1 increases production and secretion of PAO from this subcutaneous adipose tissue depot. PAO is strongly associated with insulin sensitivity, independent of age, sex, and adiposity in healthy humans [51,66], but is not associated with decreased obesity [67]. In contrast, in patients with type 1 diabetes, the concentration of circulating PAO was adversely associated with adipose tissue insulin sensitivity following a hyperinsulinemic/euglycemic clamp, but positively associated in the healthy counterparts [68]. Thus, this lipokine is correlated with improved insulin sensitivity in mice and normoglycemic humans, but appears to have a different function in a diseased population.

PAO also mediates insulin sensitivity in skeletal muscle and liver [32]. The effect of PAO on skeletal muscle insulin sensitivity was first described in FABP4/5-deficient mice [32,34]. Cao et al. [32] demonstrated that pre-treatment of C2C12 differentiated myotubes with plasma lipids extracted from *Fabp^-/-^* mice or PAO alone enhanced insulin-stimulated Protein kinase B (AKT) phosphorylation and increased glucose uptake (Figure 2E). In the liver, when the FA elongating-enzyme ELOVL6 is absent, PAO is increased and is associated with protection from hyperinsulinemia, hyperglycemia, and hyperleptinemia [69]. In parallel with the effects in muscle, PAO stimulates glucose uptake in liver and this is likely mediated by both 5’-AMP-activated protein kinase (AMPK) and Fibroblast growth factor 21 (FGF-21), which require Peroxisome proliferator-activated receptor α (PPARα) [70]. When mice were fed a high-fat diet and treated with the insulin-sensitizing drug rosiglitazone, SCD1 was quickly induced in multiple tissues including the liver, which results in an increased PAO in the cholesteryl ester, triglyceride, phosphatidylcholine, and phosphatidylethanolamine lipid fractions [71]. The effect of rosiglitazone on PAO fat-partitioning might be related to its glycemic control in the liver. Together, these studies indicate that PAO is an important contributor to peripheral tissue insulin sensitivity and glucose homeostasis (Figure 2D).

##### Palmitoleate Decreases Cellular Stress and Inflammation

MUFAs play an important role in stress and inflammation [72,73,74,75], and the consumption of a MUFA-rich diet in overweight subjects reduces the pro-inflammatory profile and, thus, leads to risk reduction for metabolic diseases [76]. Supplementation of PAO (250 µM) in differentiated 3T3-L1 preadipocytes downregulates inflammatory genes including Toll-like receptor signaling (*Tlr*), cytokine-cytokine receptor interaction, and chemokine signaling. Incubation with PAO also blocked the inflammatory hub, C-C motif chemokine ligand 5 (CCL5) [77]. Thus, PAO is effective at modulating adipose inflammation in vitro (Figure 2A).

The role of PAO in skeletal muscle stress and inflammation has been thoroughly investigated. In C2C12 myotubes, PAO delays the activation of the pro-oxidative and pro-inflammatory marker Cyclooxygenase 2 (COX-2) [78]. Moreover, conditioned media from macrophages pre-incubated with PAO blocked p38 MAPK-mediated skeletal muscle insulin resistance in C2C12 myotubes, and reverted the insulin resistant effects of palmitic acid [79]. PAO also ameliorated the pro-inflammatory effect of palmitate in L6 rat myotubes, decreased lipid accumulation in L6 myotubes [80], and decreased intra-myofibrillar lipid accumulation in obese sheep, which likely contributes to improved insulin sensitivity in skeletal muscle [81]. PAO secreted from adipose tissue decreases pro-inflammatory immune cell activation and lipid accumulation in skeletal muscle, which, in turn, contributes to the improved skeletal muscle insulin signaling and enhancement of glucose uptake (Figure 2E).

Endoplasmic reticulum (ER) stress is an important signaling hub that integrates metabolic needs with the stress responses of the cell. Importantly, PAO inhibits expression of liver ER-stress genes (*Ire-1*, *Xbp-1*, *p-eIF2*, *Jnk*, *Perk*) and decreases nuclear expression of apoptotic genes (*Chop*, *Puma*, *Bim*). This prevents apoptosis in hepatocytes [82]. PAO also reduces apoptosis in pancreatic β-cells and blocks an important arm of the ER-stress unfolded protein response (UPR) called the Protein Kinase R (PRKR)-Like Endoplasmic Reticulum Kinase (PERK) [83,84,85]. These data indicate that PAO is an important factor that protects liver and pancreatic function and modulates the ER-stress response (Figure 2B,C).

Multiple animal and epidemiological studies have associated PAO with improvements in cardiovascular health [48,49,51]. Supplementation of PAO to immune cells in both mice and humans ameliorates the development of atherosclerosis [86]. The double bond of palmitoleate, known as cis- palmitoleate or trans-palmitoleate, is essential for atherosclerosis protection. Only the cis-palmitoleate conformation reduces ER-stress and inflammation, while the trans-conformation does not [87]. The supplementation of PAO increases the rate of ER membrane-lipid remodeling in macrophages and other tissues, which prevents the lipid-mediated activation of the inflammasome and prevents activation of pro-inflammatory cells [86]. In diabetic βV59M mice, the concentration of PAO decreased in the heart [88], which may contribute to the progression of the disease and lead to metabolic and cardiac dysfunction (Figure 2D). PAO also influences vascular function by negating pro-inflammatory activation in the vascular system. PAO has a potent anti-inflammatory effect in human endothelial cells (EAHy926 cells) [89], and incubation of human endothelial cells (HUVECs) with either *t*-vaccenic acid (a PAO precursor) or *t*-palmitoleic acid decreases inflammation [90,91]. PAO prevents apoptosis of both human coronary artery endothelial cells (HCAECs) and stearate-induced apoptosis [92]. Altogether, mounting evidence demonstrates the important role of PAO on metabolic stress and inflammation, which may contribute to the enhancement of insulin sensitivity. Therefore, a strong amount of human and in vitro evidence indicates that concentration of PAO is positively correlated with improvements in inflammation and stress resilience, while concomitantly decreasing endothelial cell inflammation and immune cell activation.

#### 3.2.3. Exercise Regulation of Palmitoleate

**Figure 3:**
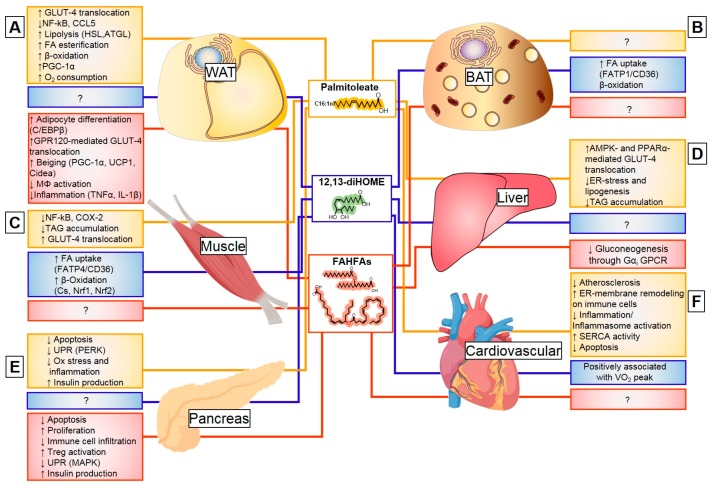
Metabolic targets of lipokines. Lipokines have diverse targets that lead to improved glucose homeostasis and metabolic health. (**A**) In white adipose (WAT), *Palmitoleate* increases glucose uptake (GLUT4), reduces inflammation (NF-kB), and increases lipolysis of lipid droplets (HSL, ATGL) to provide fuel for β-oxidation, which increases oxygen consumption. While no effects have been described for *12, 13-diHOME* in WAT, *FAHFAs* increase glucose uptake (GLUT4), adipocyte differentiation, decrease inflammation, and promote beiging. (**B**) In brown adipose (BAT), the only known effect of lipokines is for *12, 13-diHOME*, where it contributes to cold-induced adaptation by increasing the uptake of FA and enhancing β-oxidation. (**C**) In skeletal muscle, *Palmitoleate* ameliorates inflammation and oxidative stress, while decreasing intramyofibrillar TAG deposition and improving glucose uptake (GLUT4). On the other hand, *12, 13-diHOME* is a strong effector of muscle function. It is the main driver of the physiological adaptation to exercise by increasing the uptake of FA and enhancing β-oxidation. No effects are known for FAHFAs. (**D**) In the liver, the only documented effect by a lipokine is for Palmitoleate, where it increases glucose uptake, decreases stress, and reduces steatosis by downregulating lipogenesis. (**E**) Pancreatic function is improved by both palmitoleate and FAHFAs and increases insulin production. Both lipokines modulate apoptosis, oxidative stress, and the unfolded protein response (UPR). PAHSAs modulate immune cell infiltration in a model of type 1 diabetes. No effects are known for *12, 13-diHOME*. (**F**) Cardiovascular improvements are observed with both palmitoleate and *12, 13-diHOME*. Palmitoleate is associated with a reduction in atherosclerosis driven by immune-cell endoplasmic reticulum membrane lipid remodeling that results in reduced inflammation. Furthermore, the SERCA pump is improved with palmitoleate administration, which contributes to functional improvements. *12, 13-diHOME* appears to be positively correlated with cardiometabolic fitness or VO2 peak, but no studies have assessed the influence on cardiovascular function. No effects are known for FAHFAs.

Exercise is a well-established therapeutic strategy to improve glucose homeostasis [4,6,93,94,95,96,97]. Although exercise-driven adaptations to metabolic and cardiovascular health have been widely studied [4,61,94,95,96,97], little is known regarding the effect of exercise on PAO synthesis and accumulation. Exercise affects lipolysis-driven increases in circulating palmitoleate, and one study demonstrated that chronic, moderate intensity endurance exercise (treadmill running for 4 weeks) increased AT lipolysis, which released stored palmitoleate into circulation in wild-type mice [98]. In the absence of exercise-induced lipolysis (using adipose tissue triglyceride lipase deficient mice (ad-*Atgl*^-/-^)), palmitoleate was reduced in serum, which diminishes the beneficial effects of this lipokine. Specifically, exercise trained ad-*Atgl*^-/-^ mice failed to exhibit exercise-induced lipolysis, cardiac hypertrophy, and non-proliferative cardiomyocyte growth. Supplementation of palmitoleate in ad-*Atgl*^-/-^ mice restored left ventricular mass to that of wild-type mice [98] (Figure 2D), which indicated that PAO is an essential component in several of the exercise-induced benefits on metabolic and cardiovascular health. Another study demonstrated that exercise-training in mice fed a high-fat/low-carbohydrate diet decreased PAO content in epididymal WAT (eWAT) [99], likely as a result of lipolysis, and increased PAO in circulation [98]. This increase in PAO levels in circulation may be related to a decrease in WAT inflammation [55,59,77,99]. This will be the focus of future investigations (Figure 2 and Figure 3; Table 1).

Exercise also affects PAO accretion in skeletal muscle. Endurance-exercise training in rats increases accumulation of diacylglycerol (DAG) with PAO in skeletal muscle [100] (Figure 2E). Exercise-induced activation of Calmodulin-dependent protein kinase II (CAMKII) in muscle increases PAO content and reduces the saturated fatty acid content in muscle, which are both associated with insulin sensitivity [101]. Thus, exercise-training enriches PAO in the DAG and FFA-lipid portions of skeletal muscle and is likely related to improvements in insulin sensitivity (Figure 2E).

### 3.3. Oxylipins

Bioactive lipids are formed in the body from different fatty acids through the action of cyclooxygenases (COX), lipooxygenases (LOX), cytochrome oxidases (CYP450), and epoxide hydrolases (EPHX), which produce a wide range of oxylipins with far-reaching functions [119]. Oxylipins can be synthesized from ω-3 and ω-6 fatty acids and undergo different transformations to create lipid products that mediate normal physiologic functions and development of chronic diseases [119,120]. Recently, oxylipin byproducts of EPHX and LOX, specifically 12,13-diHOME [30,31] and 12-HEPE [29], have been identified for secretion from BAT in response to exercise and cold temperatures and regulate glucose, fatty acid homeostasis, and BAT thermogenesis. This makes them potential therapeutic targets.

#### 3.3.1. Discovery, Structure, and Synthesis of 12,13-diHOME

Oxylipins released from adipose tissue have been identified and their biological roles have been recently determined, including the octadecanoid 12,13-diHOME (12,13-dihydroxy-9Z-octadecenoate). This octadecanoid is a product of both the CYP450 family of enzymes that transforms linoleic acid into 12,13-EpOME, and the subsequent hydrolysis by EPHX (ER bound or cytosolic) to produce 12,13-diHOME. Both steps are required to fully convert linoleic acid (C18:2n6) into the fatty acid diol 12,13-diHOME (Figure 1). Chronic diseases such as obesity and dyslipidemia significantly reduce CYP and EPHX expression as well as their oxylipin products. In obese mice, consumption of a high-fat diet (HFD) for eight weeks alters the oxylipin profile in inguinal WAT, gonadal WAT, and interscapular BAT, and decreases both CYP (Epoxyoctadecenoic acids [EpOMEs], Epoxyeicosatrienoic acid [EETs], 19,20-Epoxydocosapentaenoic acid [19,20-EpDPE], Epoxy-octadecadienoic acids [EpODEs]), EPHX products (Dihydroxy-octadecenoic acids [diHOMEs], Dihydroxyeicosa-trienoic acids [DHET], Dihydroxy-docosapentaenoic acid [DiHDPE], and Dihydroxy-octadecadienoic acids [DiHODE]) [105]. Similarly, serum from hyperlipidemic men had reduced 12,13-diHOME, 12-HETE, 9,10-DiHODE, and 12,13-DiHODE, when compared to control subjects [106], which indicates that the metabolic derangements seen in obese or hyperlipidemic individuals can be attributed, at least in part, to the reduction of CYP and EPHX lipid products such as 12,13-diHOME.

Dietary ω-3 and ω-6 fatty acids can influence oxylipin secretion, which makes them an attractive target for the prevention and treatment of chronic diseases [108]. Dietary sources of linoleic acid, the precursor to 12,13-diHOME, include seeds and seed oils such as those from sunflower, safflower, soybean, corn, and canola, as well as whole nuts. The consumption of linoleic acid has increased over the past century by a 20-fold increase. This was observed between 1909 and 1999 [121]. Altogether, greater supplementation through diet can lead to a significant rise in circulating and tissue 12,13-diHOME levels.

#### 3.3.2. Exercise and Cold-Induced Adaptations are Mediated by the 12,13-Dihome

The first report of exercise to induce linoleic acid diols was documented in 2014 following a bout of endurance training (75 Km-cycling) in trained male cyclists [107]. In this and a follow-up study, 12,13-diHOME was speculated to be a PPAR-γ agonist and, thus, induce insulin-sensitizing effects, which are similar to other oxylipins [107,122]. Recent studies have identified 12,13-diHOME as a lipokine released from brown adipose tissue (BAT) in response to exercise [30] and cold exposure [31], which illustrates the acute induction of the epoxide hydrolases (*Ephx1-4*) leading to the production of this specific lipokine (Figure 3).

Exercise [30] and cold exposure [31] increase the production of 12,13-diHOME in BAT, which elicits adaptive metabolic responses (muscle and BAT, respectively) and improves whole-body metabolic homeostasis. An acute bout of exercise increased the concentration of 12,13-diHOME in both untrained and exercise-trained individuals, and was positively associated with cardiorespiratory fitness (VO_2_ peak). Both a single bout of exercise and exercise-training induce the expression of *Ephx1* in BAT, which increase the content of this oxylipin in serum and BAT [30]. Although several studies indicate that exercise lowers BAT activity [123,124,125], these data identify a role for exercise to increase the endocrine activity in BAT by increasing production of 12,13-diHOME, which, in turn, increases fatty acid uptake and mitochondrial fatty acid oxidation in skeletal muscle [30] (Figure 3; Table 1).

Both cold exposure (4°C) and sympathetic activation with norepinephrine increase 12,13-diHOME from BAT into the serum. Cold-induced 12,13-diHOME is acutely produced in BAT through the induction of both *Ephx1* and *2*, which increases the content of this oxylipin in serum and BAT among rodents and humans [31]. Additionally, 12,13-diHOME acts in an endocrine or autocrine manner and induces the translocation and oligemerization of the fatty acid transporter 1 (FATP1) and co-receptor CD36 that mediate fatty acid uptake. Uptake of fatty acids into BAT facilitates fuel supplementation for mitochondrial heat generation and stimulates 12,13-diHOME synthesis [31] (Figure 3).

### 3.4. Fatty Acid Esters of Hydroxy Fatty Acids

A novel class of lipids in mammals with potent antidiabetic properties—branched fatty acid esters of hydroxy fatty acids (FAHFAs)—has recently been identified [33,110,115,116,118,126]. These isomers of palmitic-acid-9-hydroxy-stearic acid (PAHSAs) confer anti-diabetic effects, promote glucose tolerance, and increase secretion of insulin and glucagon-like peptide 1 (GLP-1) [33].

#### 3.4.1. Discovery, Structure, and Synthesis of FAHFAs

FAHFAs that confer widespread anti-inflammatory and anti-diabetic effects were first identified in 2014 [33]. This first report described the identification of PAHSA isomers that originated in BAT, WAT, and liver. Structurally, PAHSA isoforms are regio-isomers, which differ in the position of the branching carbon (e.g., 5-PAHSA, 9-PAHSA, and 12/13-PAHSA, etc.). They are synthesized in response to fasting, which is decreased by the consumption of a high-fat diet, and is decreased in insulin-resistant animals and humans. Specifically, 5-PAHSA and 9-PAHSA are highly responsive to diet and constitute the biggest portion of the synthesized PAHSAs in WAT [33]. FAHFA synthesis occurs in WAT and BAT and is modulated by a carbohydrate-responsive element-binding protein (ChREBP) [33,35,111,112,114]. Adipose-specific ChREBP knockout mice display liver, muscle, and WAT insulin resistance and decreased PAHSA levels [114]. This insulin-resistant phenotype in adipose-specific ChREBP knockout mice is rescued by 9-PAHSA supplementation [114]. PAHSAs have an anti-inflammatory effect, which has been attributed to Docosahexaenoic acid (DHA) and hydroxyoctadecadienoic acid (HLA) FAHFAs, namely 9-DHAHLA, 13-DHAHLA, and 14-DHAHDHA (14-hydroxydocosahexaenoic acid) [35]. FAHFAs can be exported as non-esterified compounds or can be incorporated into TAGs, which allows their storage and enrichment in adipose tissue. FAHFA-containing TAGs are more abundant than free FAHFAs and are thought to be crucial for the regulation of their abundance and disease susceptibility [110].

The complete FAHFA biosynthetic pathway has only recently been elucidated. The proposed biosynthetic pathway for FAHFAs requires both GLUT4-mediated glucose uptake and de novo lipogenesis to generate palmitoyl-CoA under the regulation of ChREBP [127]. Overexpression of NRF2, which is a transcription factor that modulates the cellular redox state and lipid metabolism, blocked ChREBP expression and reduced the de novo synthesis of the FA (palmitate) needed for the esterification of FAHFAs. Therefore, this decreased PAHSAs. Thus, the first step of PAHSA formation requires the fine-tuning of the redox status (Nuclear Factor Erythroid 2-Related Factor 2 [NRF2]) and de novo lipogenesis (ChREBP). In addition to the synthesis of palmitate, a peroxidized membrane phospholipid (PL-OOH) is needed (in the presence of reduced glutathione [GSH]), which is then reduced by Peroxiredoxin 6 (PDRX6) and Microsomal Glutathione S-Transferase 1/3 (MGST1) into a phospholipid alcohol (PL-OH). Following the generation of the PL-OH, cleavage of this product by PDRX6 occurs, which produces hydroxyl-stearate. Lastly, the hydroxyl-stearate is further esterified to the de novo synthesized palmitoyl-CoA by the action of acyl-transferases (ACNAT1, ACNAT2, and BAAT), which generates PAHSAs [127] (Figure 1). The stereospecificity of PAHSA and FAHFA synthesis might be dependent on the acyl chains of membrane phospholipids and the peroxidases and phospholipases that cleave them, as well as the redox environment of the cell (e.g., oxidative stress, fasting, etc.). The regulation of *Pdrx6* and *Mgst1/3* by environmental factors has not been investigated.

#### 3.4.2. Methodological Identification of FAHFAs and PAHSAs

Although favorable effects of PAHSAs on glucose homeostasis and insulin sensitivity have been described, a clear consensus on the methodologies of identification, quantification, sample size, and functional effects FAHFAs is needed. Following the original publication in 2014 [33], the same group and others demonstrated the range of PAHSAs to impact glucose homeostasis [33,111,114,116,128]. However, a recent paper by Pflimlin et al. found that the acute or sub-chronic supplementation of either 5-PAHSA or 9-PAHSA, together did not improve glucose homeostasis in high-fat fed mice [129]. Further investigations indicated that each paper used different experimental and analytical methodologies that may contribute to these disparate results. A comprehensive list of the methodological differences among these studies has been previously reported [130,131]. These differences include: (1) different vehicles for in vivo PAHSA studies (PEG400/Tween80 as a vehicle with no effect on glucose tolerance [33], compared to olive oil as a vehicle that naturally contains FAHFAs) [33,130,131], (2) different mouse vendors (Jackson Labs vs. Charles River), (3) differences in the diets (chow and high-fat diet versus several high-fat diet formulations) [130,131], (4) different glucose doses used for the oral glucose tolerance test (OGTT), (1g/kg body weight versus 2 g/kg body weight), and 5) differences in dose and bioavailability of PAHSAs (1.5-fold to 3-fold versus 80-400-fold increase in serum). These data highlight the need to take into account biologically and physiologically relevant doses and their potential effects on function, which will, in turn, maximize reproducibility and discovery.

Analytical differences may also contribute to different results. An important methodological dissimilarity is aligned with the identification and separation conditions in a liquid chromatography-mass spectrometry (LC-MS) previously standardized method, with solid phase extraction, an internal standard of 13C-labeled FAHFA, and two LC-MS fragmentation monitoring steps [35,116,127,131,132,133,134,135,136] versus novel LC-MS with no solid phase extraction, an internal standard of deuterium-labeled FAHFA, and a single LC-MS fragmentation monitoring step. This showed that some of their PAHSAs were below the limit of detection [129,131]. The original report highlights the difficulty of separating FAHFAs given that the resolution achieved was unable to separate 12-PAHSA and 13-PAHSA adequately [33]. A complete list of LC-MS methodological differences can be found in Table 2 of Syed et al. [131], which sheds light on the experimental considerations for the study of PAHSAs and the complexity of the administration among in vivo systems. It is clear that PAHSAs are strong modulators of glucose tolerance and produce a profound metabolic adaptation in adipose and other metabolically-relevant tissues, but establishing standard methodologies could aid in identifying potential therapeutic targets of adipose tissue.

#### 3.4.3. Endocrine Action of FAHFAs

Insulin resistance and obesity directly impact PAHSA content in circulation and in WAT, BAT, and liver in mice and humans. Insulin resistant individuals [33] and obese mothers [133] have decreased PAHSA levels in serum and adipose tissue compared to their healthy counterparts [33,133]. Humans with impaired adipose tissue insulin sensitivity have decreased concentration of PAHSAs in their adipose tissue [113]. Administration of PAHSAs in pre-adipocytes increases their differentiation through the activation of CCAAT-enhancer-binding proteins (C/EBP) transcriptional activity [113]. Thus, insufficient levels of PAHSAs in serum and adipose tissue are correlated to obesity and diabetes and, due to their ubiquitous nature, could contribute to insulin resistance in multiple tissues (Figure 3; Table 1).

Administration of 5-PAHSA and 9-PAHSA contribute to glucose tolerance, insulin sensitivity, and immuno-modulation. Administration of PAHSAs enhances Glucagon-like peptide 1 (GLP-1) secretion and glucose uptake. PAHSAs act through a G-protein coupled receptor (GPCR)-associated pathways similar to GLP-1 [137]. PAHSAs can directly bind GPR120, which is a modulator of insulin sensitivity, in adipocytes and increase GLUT-4 translocation and glucose uptake. 9-PAHSA supplementation in 3T3-L1 adipocytes increases the expression of genes involved in ‘beiging’ of WAT, through a mechanism dependent on GPR120 [109]. Mice with adipose-specific depletion of ChREBP have impaired de novo lipogenesis and are insulin resistant, which is associated with low PAHSA levels. However, supplementation with 9-PAHSA completely restores their insulin sensitivity and reduces WAT inflammation [114]. Lastly, PAHSAs appear to coordinate adipose-liver inter-tissue communication by inhibiting WAT lipolysis and decreasing hepatic gluconeogenesis through cAMP-dependent pathway involving Gαi GPCR [117], which improves hepatic and systemic insulin sensitivity. Thus, modulation of glucose transport and inflammation amelioration appear to be mediated, at least in part, through GPCR modulation, in particular GPR120 (Figure 3).

PAHSAs also have an endocrine effect on the pancreas, which likely influences insulin sensitivity. PAHSAs bind the free fatty acid receptor 1 (GPR40) in pancreatic β-cells and negate the deleterious effects of the HF diet on pancreatic islet function [116,138]. PAHSAs increase intracellular Ca^2+^ levels in β-cells and augments GLP-1 and insulin secretion. A recent study suggests that daily oral administration of 5- PAHSA and 9-PAHSA (15 mg/Kg) in non-obese diabetic (NOD) mice delayed the onset of type 1 diabetes and reduced the overall presence of diabetes. This effect was mediated, in part, by the modulation of immune cell infiltration and ER-stress reduction, which improved glucose-stimulated insulin production and glucose tolerance. Therefore, this effect promoted β-cell survival [115]. These studies on the effects of pancreatic GPR40 amplification highlight the idea that PAHSAs may be a relevant therapeutic target for Type 1 and 2 diabetic patients.

FAHFAs also exhibit anti-inflammatory effects in WAT [114] and this function is shared by other DHA-derived FAHFA species, 9-DHAHLA, 13-DHAHLA, and 14-DHAHDHA. These lipid species are synthesized at the same concentration as other inflammation resolution factors such as resolvins and protectins (oxylipins) in adipose tissue [35]. In particular, 13-DHAHLA is an anti-inflammatory lipokine that targets immune cell activation, decreases lipopolysaccharide (LPS)-induced macrophage secretion of IL-6, TNFα, and IL-1β, increases phagocytosis, and enhances the pro-resolving process (increases 17-HDHA, protectin D1, resolving D1) [35]. While important, a direct correlation between this anti-inflammatory function and insulin sensitivity has not been established. Altogether, 13-DHAHLA may decrease adipose tissue inflammation and improve adipose tissue and immune function. Together, these studies have increased our understanding on FAHFA biology and function, including their important role as an anti-diabetic or anti-inflammatory therapeutic target, which mainly encompasses anti-diabetic and anti-inflammatory effects.

## 4. Conclusions

The rapidly expanding family of lipokines has provided substantial evidence for the complex regulation and functional plasticity in adipose tissue metabolism. Since the identification of the first lipokine more than a decade ago, several novel roles for these lipid products in inter-tissue endocrine communication have been identified. There is now a better understanding of their synthesis pathways, environmental regulation, and their roles in health and disease. Therapeutic strategies that aim to modulate systemic metabolism should recognize lipokines as a powerful tool and harness their potential as coordinators of multiple metabolic processes.

In this paper, we have summarized the vast literature on lipokines, their synthesis, regulation by environmental factors (diet, exercise, and cold exposure), and their endocrine effects, which all provide an important rationale for PAO, 12, 13-diHOME, and FAHFAs as attractive targets of novel strategies to increase insulin sensitivity, as well as muscle and liver function. Future studies will be able to elucidate additional properties of lipokines that mediate metabolic improvements, which, in turn, will inform discovery and translational potential to a clinical population. We recognize the important contribution of environmental factors to lipokine bioavailability and accumulation that expands our understanding of how the therapeutic potential of environmental factors could be harnessed. In recent decades, we have learned a great deal regarding adipose biology and its role as a secretory organ, which has led to the identification of critical homeostatic pathways where lipokines play a major role. The plasticity of lipokine function beyond anti-diabetic and anti-inflammatory mechanisms provides an enticing future for the identification of additional therapeutic targets.

## Figures and Tables

**Table 1:** Lipokine main sources and tissue targets.

Lipokine	Source	Target Tissue	References
Palmitoleate	Dietary (salmon, cod liver oil, dairy products, and macadamia oil) Endogenous synthesis (mainly WAT)	WAT	[32,43,45,52,53,55,56,59,60,65,77,98,99]
		Liver	[32,57,70,71,82,102]
		Pancreas	[58,83,84,85,103,104]
		Endothelial cells	[80,85,87,89,90,91,92,98,102]
		Immune cells	[79,85,87]
		Muscle	[32,78,81,100,101]
12,13-diHOME	Dietary (sunflower, safflower, soybean, corn, and canola oils as well as nuts and seeds) Endogenous synthesis (mainly BAT)	BAT	[30,31,105]
		Muscle	[30,106,107]
		Immune cells	[108]
FAHFAs	Dietary (olive oil, Arabidopsis thaliana, rice) Endogenous synthesis (mainly WAT and BAT)	WAT	[33,35,109,110,111,112,113,114]
		Pancreas	[115,116]
		Liver	[117]
		Intestine (colon)	[118]
		Immune cells	[35]

WAT: White adipose tissue; BAT: Brown adipose tissue; FAHFA: Fatty acid hydroxy fatty acids.
